# Healthcare Professionals' Perceptions of Future Leadership in Digital Healthcare: A Qualitative Study

**DOI:** 10.1111/jan.17035

**Published:** 2025-05-08

**Authors:** Marika Määttä, Mira Hammarén, Suvi Kuha, Outi Kanste

**Affiliations:** ^1^ Research Unit of Health Sciences and Technology University of Oulu Oulu Finland; ^2^ Medical Research Center Oulu, Oulu University Hospital University of Oulu Oulu Finland

**Keywords:** digital, healthcare, leadership, nurse, professional, qualitative study

## Abstract

**Aim:**

To describe and enhance the understanding of healthcare professionals' perceptions of future leadership in digital healthcare.

**Design:**

A qualitative descriptive study.

**Methods:**

The data were collected remotely between February and November 2022 through semi‐structured interviews. A total of 26 healthcare professionals were interviewed individually or in focus group interviews at the university hospital and university in Finland. The data were analysed using inductive content analysis.

**Results:**

Results revealed seven main categories that described the professionals' perceptions of future leadership in digital healthcare: building a future‐oriented healthcare, strengthening a digitally minded organisational culture, being interactive in a digital environment, leading sustainably in digital healthcare, leading expertise in digital healthcare, leading collaboratively in digital healthcare and using artificial intelligence in leadership in digital healthcare.

**Conclusion:**

Future leadership in digital healthcare will be about leading people in a humane way. Leaders will be at the forefront of digital solutions, sharing their expertise and enabling stakeholders' engagement. Through collaboration, future leaders will be building a future‐led digital health system.

**Impact:**

Digital healthcare is improving due to the implementation of new digital solutions and the possibility of artificial intelligence. Thus, leaders' competencies in digital healthcare need to be further developed through education and guided by policy to meet the expectations of future professionals, nurses and customers.

**Patient or Public Contribution:**

No patient or public contribution.

**Reporting Method:**

The Consolidated Criteria for Reporting Qualitative Research (COREQ) checklist was used in the reporting.

**Summary**

- What does this paper contribute to the broader global clinical community?
  ○ Leaders must consider the unique aspects of the digital environment when leading healthcare professionals, and particularly nurses, who constitute a significant portion of the healthcare workforce.
  ○ Enabling professionals and nurses to innovate and participate is crucial in developing future healthcare services.
  ○ Artificial intelligence will enhance healthcare and nursing leadership by providing information while freeing up leaders' resources.

## 1 Introduction

The digitalisation of healthcare services and organisations and artificial intelligence (AI) are constantly transforming healthcare operations and creating new conditions for leadership (Laukka, Hammarén, et al. 2022; Morse and Warshawsky 2021). COVID‐19 highlighted the challenges in healthcare, such as disruptions in service chains, difficulties in access to healthcare services and inequalities in service effectiveness, which need to be tackled by future leadership (Omaghomi et al. 2024). Future leadership in healthcare refers to a leader's ability to meet the challenges of the future through digitalisation, work cultures, sustainable working conditions, and flexible, people‐centred leadership (Vuorivirta‐ Vuoti et al. 2023). There is an increasing emphasis on people‐led leadership in the future of digital healthcare to ensure that customers continue to receive the care they need in a safe, high‐quality and timely manner (WHO 2022a).

Digital healthcare, also known as eHealth, involves the use of digital tools, information systems, and communication technologies to deliver, enhance and manage healthcare services (WHO 2022a; Al‐Jaroodi et al. 2020), including technologies such as telemedicine, electronic health records, mobile health apps and AI‐driven diagnostics. Digital healthcare aims to achieve more consistent quality, timely delivery and better availability and accessibility of treatment (Babaei et al. 2023). It also seeks to make the everyday lives of customers and the work of professionals' smoother (Kaihlanen et al. 2023). The purposeful use of digital technologies and anticipation of using health information creates value for customers, clinicians and healthcare organisations' suppliers (Dal Mas et al. 2023). However, it has been observed that an increasing number of transformation processes are unsuccessful due to poor leadership. This failure underscores the necessity for leaders to receive training, enabling them to effectively guide digital healthcare initiatives (Brommeyer and Liang 2022; Morse and Warshawsky 2021). The continuous changes in organisations enabled by digitalisation challenge education to keep up with these changes (Laukka et al. 2023). Additionally, leadership failure can result from confusion in job descriptions and leadership roles (Nurmeksela et al. 2021). Clarifying these aspects can enhance and streamline leadership, thereby improving decision‐making and the implementation of transformations (Laukka et al. 2020).

In the past, the central role of healthcare leaders was to lead clinical services. Today, however, the leader's responsibility increasingly focuses on designing, implementing and leading digital services (Morse and Warshawsky 2021). Presently, decision‐making involves interpreting and using data from clinical and administrative information systems (Al‐Jaroodi et al. 2020). The digital transformation in healthcare created by digitalisation requires competent leaders who can lead in a knowledge‐based, strategic and operational way (Brommeyer and Liang 2022). Furthermore, the successful implementation of digital solutions requires appropriate technology, sufficient financial resources and effective leadership (Burgess and Honey 2022; Vuorivirta‐ Vuoti et al. 2023). Both leaders and professionals need to understand, accept and see positively the new ways of working in digital healthcare (Gjellebæk et al. 2020; Laukka, Hammarén, et al. 2022). This necessitates the need for leaders to be educated (Brommeyer and Liang 2022) and well‐informed (Brommeyer and Liang 2022; Vuorivirta‐ Vuoti et al. 2023) to use digital solutions and lead in a digital environment.

The shift from routinised tasks to work that involves continuous learning and development (Gjellebæk et al. 2020) requires leaders to possess a variety of competencies to succeed (González‐García et al. 2021; Morse and Warshawsky 2021). While the ability to motivate and educate professionals is already a crucial aspect of effective leadership today, its importance will be further amplified in the future. Leaders will need to continuously motivate and educate professionals, such as nurses within the organisation, to utilise and apply the solutions that digitalisation enables (Laukka et al. 2023), as well as involve them in designing and implementing technology in health services (Gjellebæk et al. 2020).

## 2 Background

Digital healthcare enables the treatment of patients over distances using information and communication technology (WHO 2022b; Babaei et al. 2023). By leveraging data analytics collected through digital health systems, healthcare providers can identify ongoing health trends and patterns, allowing for more individualised and appropriate services (Al‐Jaroodi et al. 2020; Cozzoli et al. 2022). AI is also constantly playing an increasing role in customer work and administration in digital healthcare (Chen et al. 2022; Petersson et al. 2022). It affects the roles of healthcare professionals in organisations (Petersson et al. 2022), which also involves new kinds of needs for leadership (Laukka, Pölkki, et al. 2022). Leaders have a crucial role when implementing AI in practice, as they have expertise in healthcare's peculiarities, such as customer safety and ethical considerations (Ross et al. 2024). The utilisation of AI can enhance leaders' decision‐making and operational efficiency, but it requires specific training (Gonzalez‐Garcia et al. 2024). While it fosters innovative leadership and professional development, its implementation also brings about ethical concerns (Ross et al. 2024) and resistance to change (González‐García et al. 2021).

The changes in society and working life in the 21st century have also changed the conditions identified for leadership, and unnecessary hierarchy and bureaucracy have gradually been abandoned (Gjellebæk et al. 2020). However, healthcare leadership has been criticised for being occupationally focused, fragmented and overburdened (Terkamo‐Moisio et al. 2024). The healthcare sector is highly expert‐based and heavily relies on competencies, which are emphasised between different professional groups and leaders (Terkamo‐Moisio et al. 2024). Future generations entering the workforce will influence leadership structures in healthcare by bringing new expectations and values. They advocate for more collaborative and inclusive leadership styles (Vuorivirta‐ Vuoti et al. 2023).

In the future, healthcare leadership needs to strengthen digital transformation, invest in workforce development, promote the development of expertise and support an organisational culture that facilitates adaptation to changes (Omaghomi et al. 2024). Future leaders are expected to prioritise sustainable working conditions, well‐being at work and the attractiveness of the healthcare industry (Vuorivirta‐ Vuoti et al. 2023). Open communication has increased job satisfaction and commitment to the organisation among professionals, which necessitates paying attention to leaders' communication competencies (Fowler et al. 2021). The new generations, Millennials and Generation Z, possess greater digital competence, which can shape their future expectations for leadership (Brommeyer and Liang 2022). The challenge of future leadership in digital healthcare is to ensure good interaction and communication between people when working via digital solutions (Vuorivirta‐ Vuoti et al. 2023).

Healthcare is undergoing significant reforms and changes (Al‐Jaroodi et al. 2020), necessitating continuous renewal of leadership and forecasting future leadership (Gjellebæk et al. 2020). The achievement of organisational goals can be ensured by investing in the organisation's most critical asset: its professionals (Juchnowicz and Kinowska 2022; Oleksa‐Marewska and Tokar 2022). By anticipating the conditions created by future leadership, the aim is to meet the expectations created for it more appropriately, especially when the new generations are entering the workforce (Vuorivirta‐ Vuoti et al. 2023). Understanding and anticipating future leadership also facilitates planning leadership‐related education and competence.

Future leadership in healthcare has previously been studied from the leaders' perspective, mainly in hospital settings (Gjellebæk et al. 2020; Vuorivirta‐ Vuoti et al. 2023) and the competencies required in leadership in digital healthcare (Laukka, Pölkki, et al. 2022; Morse and Warshawsky 2021). There are limited studies on future leadership in the post‐pandemic era, particularly in the context of digital healthcare, which has expanded significantly. This descriptive qualitative study contributes to healthcare professionals' and, in particular, nurses' perceptions of future leadership in digital healthcare. Understanding these perceptions will help prepare leaders to navigate the challenges and opportunities presented by digital healthcare.

## 3 The Study

### 3.1 Aim and Research Question

This study aimed to describe and enhance the understanding of healthcare professionals' perceptions of future leadership in digital healthcare.

The research question was ‘What are healthcare professionals' perceptions of future leadership in digital healthcare?’

### 3.2 Design

The study used a qualitative descriptive design to describe healthcare professionals' perceptions of future leadership in digital healthcare (Polit and Beck 2021). The qualitative design was chosen because the available studies on future leadership in digital healthcare are limited, and there is a need to understand the phenomenon from the perspective of healthcare professionals, and particularly nurses, who represent most healthcare professionals. The Consolidated Criteria for Reporting Qualitative Research checklist (Supporting Information S1) was used to ensure comprehensive and transparent reporting of the study (Tong et al. 2007).

### 3.3 Participants and Recruitment

The participants (*n* = 26) were all healthcare professionals (e.g., nurses, public health nurses, occupational health nurses) with a background working at a Finnish university hospital (*n* = 12) and healthcare professionals (*n* = 14) studying health management science at a Finnish university. The university hospital employs approximately 7500 healthcare professionals across 35 medical specialities and extensively uses digital health services and technological solutions. These services include digital pathways, virtual hospital systems, digital Health Village platforms, remote clinics, robotics and other technological solutions. The university collaborates closely with the university hospital in teaching and research. With 14,200 students and 3800 employees, the university educates healthcare, nursing and medical technology experts and managers.

Two distinct recruitment strategies were employed. The healthcare professionals from the university hospital were recruited by using purposive sampling (Polit and Beck 2021). A contact person from the university hospital offered a list of potential participants and distributed the invitations. After only a few professionals declared their willingness to participate, a snowball sampling approach was used to recruit more participants (Polit and Beck 2021). The professionals from the university were recruited by using purposive sampling (Polit and Beck 2021). They were pursuing a master's degree in health management science and were enrolled in a healthcare leadership course at the time of data collection. The inclusion criteria for participation were experience as a healthcare professional and adequate Finnish language skills. At the beginning of the study, the participants were informed by the teacher responsible about the voluntary nature of their participation. The participants indicated their willingness to participate to the teacher responsible, who then forwarded the information to the researchers who conducted the interviews.

### 3.4 Data Collection

The data were collected through semi‐structured interviews. The experienced research group constructed and developed two separate interview guides, one for individual interviews and one for focus group interviews, based on previous studies on leadership in digital healthcare (Gjellebæk et al. 2020; Laukka, Hammarén, et al. 2022; Laukka, Pölkki, et al. 2022; Sharpp et al. 2019). The interview guides included common themes and clarifying questions concerning future leadership in digital healthcare (Table 1). The interview guides were pretested with two healthcare professionals separately. The pretest required only minor adjustments, with the number of questions reduced and duplication removed. The interviews conducted during the pretest were included in the final data.

**TABLE 1:** Questions for individual and focus group interview guides concerning future leadership in digital healthcare.

Individual interview questions for professionals working in the university hospital
Leadership in digital health services How do you feel about leadership using digital tools (such as email or Teams)? How do you think leaders are leading digital healthcare services and their use? What are your expectations for a leader in the digital health services? What kind of leadership do you want in the future? What do you expect from a leader in the future?
Artificial intelligence in healthcare How would you describe the use of artificial intelligence in your work? What kind of experiences do you have with artificial intelligence in healthcare? What kind of thoughts does artificial intelligence evoke in you? What do you envision as the future role of artificial intelligence in healthcare?

Focus group interview questions for healthcare professionals studying at the university
Leadership in digital healthcare services What is the nature of leadership in digital healthcare services? How do you think leaders are leading digital healthcare services and their use? What kind of specific leadership is expected when operating digital healthcare? What kind of leadership is desired now and in the future?
Utilisation of digital methods in leadership How do you feel about leadership using digital tools (such as email or Teams)? What digital tools have been used in leadership in your work?
Artificial intelligence's opportunities in leadership What thoughts do you have on artificial intelligence and its application in healthcare leadership? What role will artificial intelligence play in future healthcare?

Participants' background information (Table 2) was collected using a Webropol survey (Webropol 2025) to ensure a flexible and consistent collection of background information from all interviewees. The survey was conducted simultaneously as the information letter was sent to participants before the interviews.

**TABLE 2:** Participants' background information.

Variable		Working in the university hospital (*n* = 12)	Studying at the university (*n* = 14)
Gender	Female	11	10
	Male	1	4
Educational level	Master's degree or higher	3	3
	Bachelor's degree or lower	9	11
Organisation	Public university hospital	12	—
	Primary healthcare (public)	—	3
	Specialised care (public)	—	3
	Private healthcare	—	4
	Other^a^	—	4
Work task	Nurse	7	6
	Therapist	4	2
	Project/expert work	1	3
	Leadership roles	—	3
Age (years) mean (range)		47.4 (SD: 12.2), range: 27–63	36.4 (SD: 7.4), range: 28–52
Work experience (years) mean (range)	In current work	8.0 (SD: 7.2), range: 1.0–22.0	3.7 (SD: 3.3), range: 0–10
	In healthcare	20.0 (SD: 12.7), range: 2.0–38.0	7.8 (SD: 6.0), range: 0–24

^a^ Social care, research organisation and community care.

The data collection was conducted in two parts. First, semi‐structured interviews of healthcare professionals working at the university hospital (*n* = 12) were conducted between February and May 2022 by one researcher (PhD student, female). Second, data were collected by two researchers (PhD students, both females) in November 2022 through focus group interviews with healthcare professionals studying at the university (*n* = 14). All researchers collecting the data were trained beforehand to ensure that the interviews were conducted in accordance with the unified guidelines.

Interviews were conducted and recorded remotely via Microsoft Teams (Microsoft Corporation 2023a). At the beginning of the interviews, all participants were informed of the study's aim, the interview questions, the voluntary nature of their participation in the research, and their right to withdraw from the study. They were offered the opportunity to comment further on the topics discussed at the end of the interviews. The interviews were conducted during participants' regular working or studying hours. Only the interviewees and one interviewer were present during the interviews. None of the interviewees discontinued their participation during the study. Data collection was terminated when it was interpreted that the same themes began to recur in the interviewees' responses, indicating data saturation (Kyngäs et al. 2020). The average duration of the individual interviews was 60 min (range, 48–73 min), and for the focus group interviews, it was 84 min (range, 69–94 min).

### 3.5 Data Analysis

Inductive content analysis was employed as the data analysis method because knowledge about the phenomenon is limited and fragmented, particularly regarding healthcare professionals' and nurses' perceptions of future leadership in digital healthcare (Kyngäs et al. 2020). The data from individual and focus group interviews were analysed as a single dataset. The research question guided this inductive content analysis, and answers to the research question were sought from the data. The recorded interviews were transcribed and totalled 309 pages, formatted in 12‐point Times New Roman font with 1.5 line spacing. After the data were obtained in a written form, the analysis proceeded to the data reduction phase (Kyngäs et al. 2020). The unit of analysis was a meaning describing future leadership in digital healthcare. At the beginning of the analysis, the data were read several times to gain a deeper understanding, and the first author tabulated the original expressions. Simplified expressions (*n* = 560) were then formed from the original expressions. These simplified expressions were grouped into subcategories (*n* = 78), upper categories (*n* = 22), and finally, main categories (*n* = 7) based on content similarities. The analysis was done with Microsoft Excel (Microsoft Corporation 2023b).

### 3.6 Ethical Considerations

The study was conducted in accordance with the criteria of research integrity at all stages of the process (Finnish National Board on Research Integrity (TENK) 2023). Approval of conducting the study was obtained from the participating organisations, which considered the ethical aspects of the study. The ethics committee's approval was not required for this study because it did not involve physical harm to participants, did not involve sensitive data and only involved adult participants (Finnish National Board on Research Integrity (TENK) 2023). Before participating, all participants were informed about the study and the use of the data. Participation was voluntary, and informed consent was obtained both electronically through Webropol software (Webropol 2025) and verbally at the beginning of the interview. Participants were informed that they could withdraw from the study at any time and had the right to cancel their participation (World Medical Association (WMA) 2013). The interviews were audio‐recorded with the participants' permission. The collected data were stored and handled carefully, in accordance with the General Data Protection Regulation (European Union 2016).

### 3.7 Rigour

The study's trustworthiness was assessed using the criteria of credibility, dependability, confirmability, authenticity and transferability (Kyngäs et al. 2020). Credibility was established through purposive sampling, based on the study's inclusion criteria, to ensure that participants had relevant experience with the study phenomenon and that data saturation was achieved. The data analysis was conducted by one author (M.M., female, MSc), with guidance sought from three other authors (M.H., female, MSc, S.K., female, MSc, O.K. female, professor). The dependability and confirmability of the inductive data analysis were also ensured through coding consistency checks and by revisiting the original data multiple times during the analysis. To ensure reflexivity, the researchers in the research group consciously examined their own subjective perspectives on the research phenomenon during data collection and analysis, identifying how this subjectivity could impact the interviews. None of the researchers had a prior relationship with the interviewees. To assess the transferability of the findings, relevant contextual information about the study setting and participants was provided. Direct quotations were included to ensure authenticity, illustrating the connection between the findings and data. More direct quotations for each main category are presented in Table S1.

## 4 Findings

Participants' background factors are presented in Table 2. The mean age of professionals working in university hospitals was 47 years, while those studying at the university were 36 years old. The mean work experience in healthcare was among the professionals working in university hospitals for 20 years, and among the professionals studying at university for approximately 8 years. Among all participants, half worked as a nurse, a quarter as a therapist, and the rest worked on a project or expert work or in a leadership role.

**FIGURE 1:**
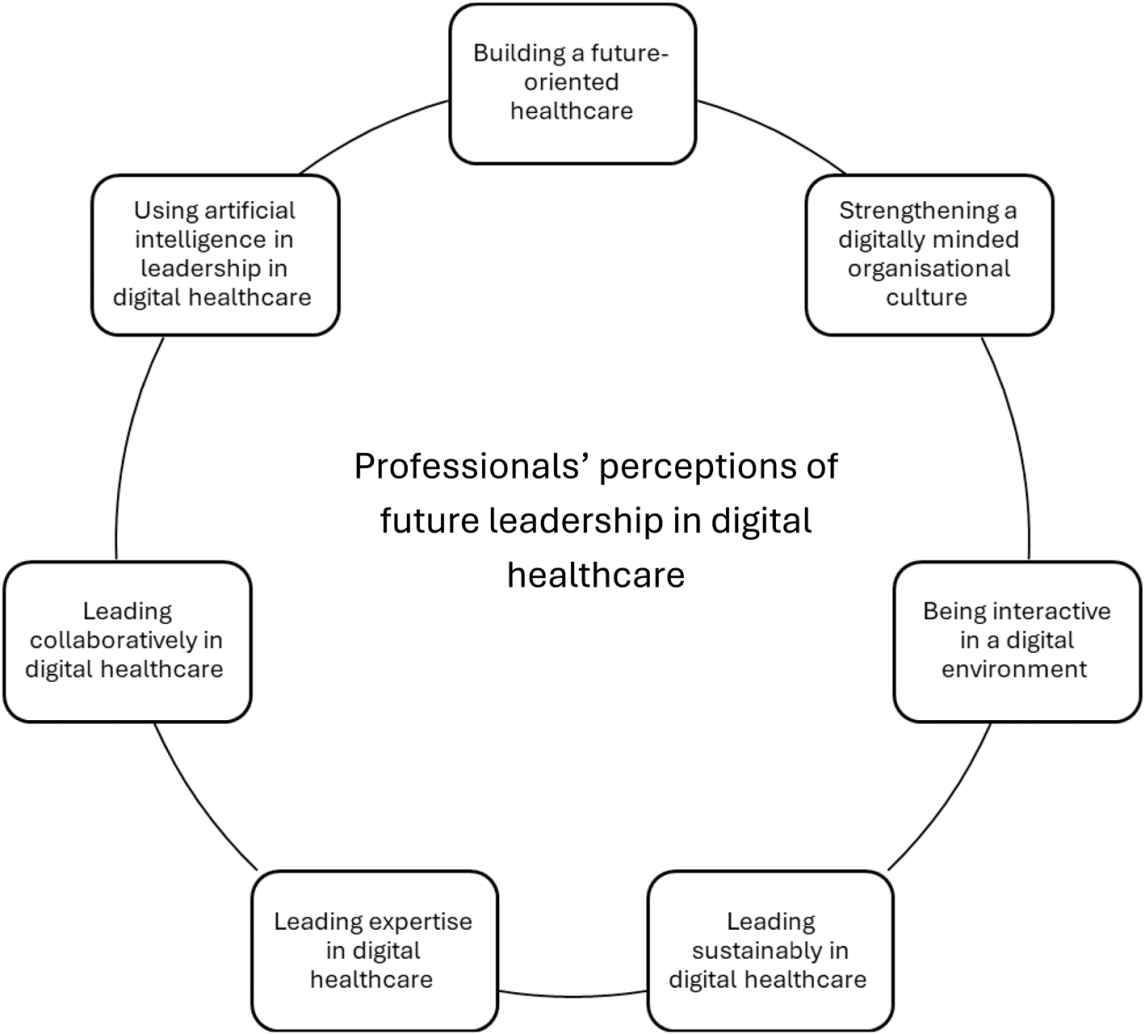
Professionals' perceptions of future leadership in digital healthcare.

Based on the analysis, seven main categories emerged, representing professionals' perceptions of future leadership in digital healthcare: (1) building a future‐oriented healthcare, (2) strengthening a digitally minded organisational culture, (3) being interactive in a digital environment, (4) leading sustainably in digital healthcare, (5) leading expertise in digital healthcare, (6) leading collaboratively in digital healthcare and (7) using AI in leadership in digital healthcare (Table 3, Figure 1).

**TABLE 3:** Professionals' perceptions of future leadership in digital healthcare.

Subcategories (*n* = 78)	Upper categories (*n* = 22)	Main categories (*n* = 7)
Managing information	Leading with information in future digital healthcare	Building a future‐oriented healthcare
Using information in decision‐making		
Analysing available information		
Knowledge management is the duty of the top management		
Understanding of the groundwork of the organisation's units	Developing digital services to meet operational demands	
Understanding the processes of digital services		
Developing services in digital healthcare		
Preparing changes in advance		
Implementing controlled changes		
Contributing to the preparation of the organisation's strategy		
Enabling innovative activity	Being innovative in digital healthcare	
Innovativeness in developing digital services		
Modernising leadership to meet changing circumstances		
Managing the use of digital solutions in leadership	Requiring digital competence when leading in digital healthcare	
Managing the use of technology in leadership		
Ensuring leaders' competence in digital leadership	Being a competent leader in digital healthcare	
Ensuring leaders' competence in changes		
Having interpersonal skills in the digital working environment		
Being determined in decision‐making in digital leadership		
Leading changes with a positive attitude	Fostering digital mindset through leaders' attitudes in organisations	Strengthening a digitally minded organisational culture
Leading by example in digital healthcare		
Committing to the use of digital solutions		
Allocating responsibilities to professionals to support their expertise	Creating an atmosphere to support professionals' expertise development	
Strengthening the learning atmosphere		
Engaging professionals in the adoption of digital solutions by justifying the benefits		
Promoting knowledge sharing through a leader's digital mindset		
Harnessing the talents of the work community to promote knowledge sharing		
Understanding the impacts of digital solutions on professionals	Creating an atmosphere of digital thinking in the organisation	
Understanding the impacts of digital solutions on customers		
Strengthening the digital mindedness in digital healthcare		
Having communication competence	Supporting operational change through on‐time communication	Being interactive in a digital environment
Communicating on‐time of changes		
Leading interactively during changes		
Communicating clearly in digital healthcare	Communicating effectively in a digital environment	
Communicating systematically in digital healthcare		
Ensuring the transmission of information in a digital working environment		
Interacting via digital solutions		
Promoting digital competence through communication		
Ensuring the effectiveness of internal communication within the organisation		
Maintaining openness in the interaction in a digital environment		
Ensuring two‐way communication between leaders and professionals	Utilising communication in decision‐making	
Making decisions interactively		
Providing appropriate information		
Utilising information as part of leading well‐being at work	Supporting professionals' wellbeing at work in digital healthcare	Leading sustainably in digital healthcare
Observing professional's work autonomy and workload		
Flexibility in leading professionals	Promoting humanity through leadership	
Considering individuality in leadership		
Taking professionals into account in leadership		
Treating professionals equally		
Considering the effects of changes on staff	Supporting professionals in the digital transformation	
Ensuring good accessibility of leaders		
Being present when leading changes		
Directing adequate orientation resources to the change process	Ensuring adequate resources for change processes	
Directing adequate training resources to the change process		
Directing adequate working time resources to the change process		
Ensuring adequate resources for the deployment of digital services		
Ensuring the knowledge of professionals	Supporting professional's digital competence	Leading expertise in digital healthcare
Assessment of competences		
Developing professionals' competence in digital healthcare		
Supporting professionals' digital skills in changing digital healthcare		
Valuing the competence of different generations	Leading different generations in digital healthcare	
Strengthening intergenerational interaction		
Strengthening intergenerational work		
Supporting the competence of different generations of professionals		
Taking different perspectives into account as part of decision‐making when leading different generations		
Leading effective location‐independent teams	Strengthening teamwork in digital working environment	Leading collaboratively in digital healthcare
Forming a team in digital healthcare		
Harnessing professionals' expertise in decision‐making	Enabling the inclusion of professionals and customers in digital healthcare	
Consultation of staff as part of the decision‐making process		
Ensuring the involvement of different levels of the organisation in decision‐making		
Involving customers in decision‐making		
Ensuring effective cooperation at different levels of leadership in the organisation	Developing together in digital healthcare	
Leveraging collaboration in digital healthcare		
Using artificial intelligence‐generated information for leadership	Understanding the potential of using artificial intelligence for leadership in digital healthcare	Using artificial intelligence in leadership in digital healthcare
Utilising artificial intelligence when leading human resources		
Using artificial intelligence to free up resources for interactive human resource management		
Recognising the risks of diminishing human leadership when using artificial intelligence	Identifying concerns about the impact of using artificial intelligence on leadership	
The uncertain potential of artificial intelligence in leadership		

### 4.1 Building a Future‐Oriented Digital Healthcare

Building a future‐oriented healthcare included five upper categories: leading with information in future digital healthcare, developing digital services to meet operational demands, being innovative in digital healthcare, requiring digital competence in leading digital healthcare and being a competent leader in digital healthcare.

Professionals perceived *leading with information in future digital healthcare*, particularly as managing and analysing available information and using information in decision‐making. Knowledge management was expressed primarily as a duty of the top management, and it can be used when implementing future changes into practice. As future leaders have access to a wealth of information in different systems that they need to use in their work and decision‐making, it is essential for leaders to possess the competence to manage that information. By analysing and using the information available, it is possible to consider different perspectives and be responsive to develop processes that make them smoother and more effective.

According to professionals, *developing digital services to meet operational demands* requires future leaders to understand the groundwork of the organisation's units and the processes of digital services. Development has its peculiarities when working in future digital healthcare, as it is essential to consider the suitability of services for different user groups, which requires identifying the groups that will use the services. The professionals also noted the importance of considering the holistic view when treating a customer in digital healthcare rather than just one part of the process. Future leaders' interest in using digital solutions was seen as essential. Preparing changes in advance and implementing them in a controlled way were considered vital in the future. It was also highlighted that future leaders contribute to preparing the organisation's strategy by thinking about the long‐term development of digital healthcare.

Leaders need to understand the context in which digital solutions are applied. Otherwise, they will be fragmented here and there and will not serve the core mission. (Interviewee 2)

*Being innovative in digital healthcare* was an expectation for future leaders by professionals. The analysis revealed that future leaders are expected to enable innovative activity in organisations. Courage was emphasised in decision‐making and developing new approaches in the future. It was seen as essential that future leaders enable innovative solutions to make operations more effective. The leaders were also expected to allow innovativeness in developing digital services in the future. It was seen that future leaders need to enable debate and innovation on digital services. Professionals also recognised the importance of modernising leadership to meet changing circumstances in future digital leadership.

Professionals perceived that *requiring digital competence when leading in digital healthcare* is essential for the effective use of digital solutions in future leadership. It was seen that leadership via digital solutions is different from traditional leadership. The analysis revealed that when future leaders have advanced competence in using a wide range of digital solutions for leadership, they are better equipped to implement different digital solutions. The analysis also revealed that future leaders are expected to manage the use of technology in leadership. Mastering the use of technology and understanding the technology used in the professionals' daily work are considered vital as they help support professionals in problem‐solving situations.

This leadership in a digital environment, I think it also requires a whole new set of problem‐solving skills, how to deal with things remotely, how to solve things. (Focus group 2, Interviewee 2)

*Being a competent leader in digital healthcare* was perceived as crucial for the future leadership of digital healthcare. It was highlighted that future leaders need to be trained in digital leadership. Future leaders were expected to be able to identify their own strengths and development opportunities. Professionals also considered it essential to ensure future leaders' competence in leading changes. The analysis revealed that strong interpersonal skills are required from future leaders in digital working environments. In this context, managing interpersonal skills was seen differently compared to more traditional leadership. For example, showing empathy through digital solutions needs to be mastered, according to the professionals. It was seen that future leaders are expected to be determined in digital leadership, and the organisation's strategy will guide their work. The professionals considered that with determination, the team can be committed to the organisation's common goals in the future.

### 4.2 Strengthening a Digitally Minded Organisational Culture

Strengthening a digitally minded organisational culture included three upper categories: Fostering a digital mindset through leader's attitudes in organisations, creating an atmosphere to support professionals' expertise development and creating an atmosphere of digital thinking in the organisation.

The professionals expressed that *fostering a digital mindset through leaders' attitudes in organisations* is playing a key role in the future of digital healthcare. According to the professionals, future leadership requires a positive attitude that sees digital solutions as an opportunity rather than a challenge. It was highlighted that future leaders need to be change‐minded and motivated to reform healthcare. The analysis revealed that leaders' exemplary behaviour is reflected in professionals and supports them in adapting to digital solutions. Therefore, it was also important that future leaders are committed to using digital solutions when leading in digital healthcare.

It is essential that the leaders are committed to the use of these digital platforms and that they are leading by example. (Focus group 2, Interviewee 3)

The analysis showed that *creating an atmosphere to support professionals' expertise development* can be strengthened by allocating responsibilities to professionals to support their expertise. Strengthening the learning atmosphere in future digital healthcare was considered vital. It was seen that engaging professionals in adopting digital solutions can be strengthened by justifying the benefits of the solutions and the possibilities they enable in the future. Promoting knowledge sharing in digital healthcare through the digital mindset of future leaders and harnessing the talents of the work community to promote knowledge sharing were considered vital.

Professionals perceived that *creating an atmosphere of digital thinking in the organisation* requires future leaders to understand the impacts of digital solutions on staff and customers when leading in digital healthcare. It was seen that it makes it easier for future leaders if they are familiar with the services where digital solutions are included and have a holistic view of the services. As processes change with digital solutions, it was highlighted that future leaders need to consider the impacts on the work of professionals and consider them when designing work to keep professionals' positive minds towards changes. As the services change, it was important for future leaders to ensure that the operationality of services continues to meet customers' needs. Hence, professionals are more eager to use the new concepts developed. The analysis revealed that future leaders can strengthen digital mindedness in the digital environment by encouraging and building professionals' confidence. According to the professionals, it was also necessary to ensure that the applications used are fit for purpose.

Leaders must encourage employees and instill belief and confidence in their abilities and learning potential. In other words, to support the experience of capability. (Focus group 3, Interviewee 3)

### 4.3 Being Interactive in a Digital Environment

Being interactive in a digital environment included four upper categories: supporting operational change through on‐time communication, paying attention to the specifics of communication in digital healthcare, communicating effectively in a digital environment and utilising communication in decision‐making.

*Supporting operational change through on‐time communication* was seen as communicating changes on time and leading interactively during a change in digital healthcare. Leaders of the future were expected to communicate about changes in advance. Professionals thought that interactivity is emphasised when leading change in the digital environment in the future. They also perceived that the discussion about the different impacts of changes should be open, and future leaders should encourage dialogue. The analysis revealed that future leaders need to have communication competence when leading in changes.

The leader needs to have good communication competence to make things work. (Focus group 2, Interviewee 2)

According to the professionals, *communicating effectively in a digital environment* was seen as communicating clearly and systematically. Future leaders are expected to ensure the transmission of information in digital healthcare. It was considered necessary for future leadership in digital healthcare to ensure that the shared information reaches the right people through effective interaction. Professionals emphasised the need for leaders to focus on the specifics of communication in digital healthcare, as interacting via digital solutions is seen as a future leadership trend. Also, promoting digital competence through communication in future leadership was highlighted by the professionals. Ensuring the effectiveness of internal communication within the organisation by future leaders was considered vital. The analysis revealed that maintaining openness in the interaction and ensuring the transmission of information in digital healthcare was seen as essential. The professionals perceived that it could be a challenge to ensure in the future that people have access to the information they need when leading via digital solutions.

When leading remotely in a meeting, how many employees actually listen? How many then structure this information and actually put it into practice? Even in these cases, the leader's attention and initiative have to be proactive in terms of interaction and asking questions via remote solutions. (Focus group 4 Interviewee 3)

*Utilising communication in decision‐making* in future was seen as ensuring two‐way communication between leaders and professionals. Making decisions interactively and providing appropriate information in digital healthcare in the future were considered vital. The professionals perceived that in future leadership, the role of middle management is to communicate perceptions between the organisation's top leaders and professionals. The analysis revealed that, as a future leader in digital healthcare, relevant information is expected to be distributed in a targeted manner. Otherwise, the overwhelming amount of available information will make it challenging to identify what is most pertinent to one's work.

### 4.4 Leading Sustainably in Digital Healthcare

Leading sustainably in digital healthcare included four upper categories: supporting professionals' well‐being at work in digital healthcare, promoting humanity through leadership, supporting professionals in digital transformation and ensuring adequate resources for change processes.

The analysis revealed that *supporting professionals' well‐being at work in digital healthcare* was seen as utilising information as part of leading well‐being at work. Future leaders are expected to use innovations that measure workload to support leading well‐being at work. With the use of different innovations, future leaders can get data about the professionals' work and are expected to exploit it when leading in digital healthcare. It was considered that professionals' well‐being at work in digital healthcare could be supported by observation of professionals' work autonomy and workload. Future leaders are expected to assess the workload distribution across the workforce critically, but working in a digital environment does not make it easy because interacting via digital solutions does not convey the essence and emotions of a professional in the same way as meeting face‐to‐face.

With this digitalisation in mind, workload measurement innovations might be used more in future leadership. (Interviewee 6)

*Promoting humanity through leadership* was seen as flexibility in future leadership. Flexibility in leadership was requested when planning the timing of work and adapting tasks to the level of professional competence in the future. According to the professionals, individuality in future leadership was highlighted. It was seen, for example, that in the future leadership in digital healthcare, there is a need to allow more individual working times for the professionals. In future leadership, more attention needs to be paid to professionals, and future leaders need to take professionals into account when working in digital healthcare. The analysis revealed that professionals were expected to be treated as individuals with their own backgrounds rather than merely as employees, among others. Being treated equally was highlighted by the professionals, and it was seen as increasingly important in the future as the workforce becomes more multicultural.

There must be flexibility in leadership, which means understanding the different starting points and the different competence requirements and understanding that there are very different competence levels, and then leaders need to be flexible in different ways for different people. (Focus group 3 interviewee 2)

One of the ways of *supporting professionals in digital transformation* was identified in the analysis as considering the effect of changes on staff. Ensuring the accessibility of leaders was also considered vital in future leadership to support professionals in transformation. Future leaders were also expected to be present when leading changes in digital healthcare. Professionals perceived that it is essential to meet face‐to‐face with professionals occasionally, not just remotely.

*Ensuring adequate resources for change processes* in future was seen as directing sufficient orientation, training and working time resources to the change processes. Directing adequate orientation resources to the change process in future leadership in digital healthcare was seen as necessary. According to professionals, a successful orientation smoothens the work of future professionals and increases their well‐being at work. Future leaders were expected to allocate adequate training resources for change processes. Providing adequate training resources in the future was seen as essential to meet the competence needs of professionals. The analysis revealed that with adequate working time resources for change processes, future leaders were enabling professionals to adopt new working methods. Ensuring adequate resources for the deployment of digital services was considered vital in future leadership so that professionals can adopt new digital solutions and take them into their daily work in digital healthcare.

### 4.5 Leading Expertise in Digital Healthcare

Leading expertise in digital healthcare included two upper categories: supporting professionals' digital competence and leading different generations in digital healthcare.

*Supporting professionals' digital competence* involves ensuring their knowledge of digital healthcare. It was seen that as digital healthcare evolved, professionals' digital competence needed to be supported by future leaders. Assessment of professionals' competence in digital healthcare by future leaders was considered significant. Professionals perceived that, in the future, it would be essential to recognise competence needs when leading through changes. The analysis revealed that developing professionals' competence in digital healthcare through future leadership was necessary. Professionals' competence development can be achieved in multiple ways, such as by sharing topical information and fostering a supportive learning atmosphere as a future leader. Future leaders were expected to support professionals' digital skills in changing digital healthcare. According to the professionals, the digital skills needed when working in digital healthcare can be supported innovatively through training and the presence of a leader to support in solving technical problems.

It is the role of future leaders to ensure both their own competence and the competence level of those they lead to ensure the successful implementation of new operation models. (Focus group 2, Interviewee 1)

The analysis revealed that *leading different generations in digital healthcare* involves valuing the competencies of each generation. Professionals perceived that by assessing jobs on a more individual basis, future leadership could address the competence gap between different generations. It was also seen as a means to strengthen intergenerational interaction. Strengthening intergenerational work was considered important in future leadership. It was considered that ensuring adequate support for the competencies required for the job can support intergenerational interaction in future leadership. The professionals perceived that leading different generations in digital healthcare also involves supporting the competencies of different generations of professionals in the future. Considering different perspectives in decision‐making when leading different generations was highlighted as a key aspect of future leadership in digital healthcare.

The leader can demonstrate to the work community that we all have an important role to play and how we can be supported. (Interviewee 3)

### 4.6 Leading Collaboratively in Digital Healthcare

Leading collaboratively in digital healthcare included three upper categories: strengthening teamwork in digital working healthcare, enabling the inclusion of professionals and customers and developing together in digital healthcare.

The analysis revealed that *strengthening teamwork in a digital working environment* was seen as leading to effective location‐independent teams in the future. According to the professionals, teamwork can be made effective by appropriately using digital solutions. It was also seen that future leaders are in a key role when forming a team in digital healthcare. Maintaining a sense of belonging while working in a digital environment was vital in future leadership.

It adds a new dimension to leadership in that the people being led are not physically present. (Focus group 1, Interviewee 4)

The professionals highlighted that *enabling the inclusion of professionals and customers in digital healthcare* was identified as harnessing the professionals' expertise in decision‐making in future leadership. It was also seen as consulting staff as part of the decision‐making process. The involvement of professionals in the development of services and utilisation of their expertise to support decision‐making was seen as important in future leadership. Ensuring the involvement of different levels of the organisation in future leadership was also considered crucial. Professionals highlighted the consultation of the various levels of the organisation to identify common development needs. They also perceived that involving customers in decision‐making in digital healthcare is vital for future leadership. Ensuring that customers are involved in the development of services was emphasised for the future.

The analysis revealed that *developing together in digital healthcare* ensures effective collaboration at different levels of leadership in the organisation. The professionals perceived that unnecessary hierarchy should be avoided. Future leaders were expected to leverage collaboration in digital healthcare when developing together in digital healthcare. According to the professionals, collaboration requires networking skills from the future leader and the ability to work with different stakeholders.

Traditionally, we have been led a bit hierarchically and, in a top, ‐down style. But in the digital environment, I think it is especially important to learn how to lead more from the bottom up. (Focus group 2, Interviewee 1)

### 4.7 Using Artificial Intelligence in Leadership in Digital Healthcare

Using AI in leadership in digital healthcare included two upper categories: understanding the potential of using AI for leadership in digital healthcare and identifying concerns about the impact of using AI on leadership.

The analysis revealed that *understanding the potential of using AI for leadership in digital healthcare* in future was perceived as beneficial. Its use in planning was regarded as a key aspect of the future by professionals. Utilising AI in human resources management was considered an integral part of future leadership. It was also seen as creating opportunities for enhanced personnel leadership. In particular, the use of data collected by AI systems was viewed as enabling more equal treatment of professionals. AI was not seen as a future replacement for leaders. Professionals perceived that using AI in future leadership frees up leaders' resources for interactivity and human resources management, which professionals have been longing for from leaders.

Ideally, if artificial intelligence could save leaders time on things like shift planning, they would have more time to spend with employees and do other leadership‐related tasks. (Focus group 1, Interviewee 1)

*Identifying concerns about the impact of AI on leadership* was seen as recognising the risks of diminishing human leadership when using AI in future leadership in digital healthcare. The flexibility and more personalised approach in leadership and service of customers that were seen as key themes in future leadership in digital healthcare were seen diminishing in using AI‐generated knowledge by the professionals. The analysis revealed that the potential of AI in digital healthcare leadership was seen as uncertain. Professionals perceived healthcare leadership to be more human‐centred and less routine compared to other sectors, where AI can replace more aspects of leadership.

Is it then the case that the individual needs of the individual employee are forgotten? … It is precisely humanity that may suffer when artificial intelligence is used in future leadership (Focus group 2, Interviewee 2).

## 5 Discussion

This study provides novel insights into the future of leadership in digital healthcare, as perceived by professionals. In the future, leadership demands the bold adoption of new innovations, operating models and digital solutions. These innovative approaches are essential to addressing the evolving healthcare needs closely tied to broader societal changes, such as leveraging digital transformation to improve health outcomes (WHO 2022a). These professionals' perceptions add new perspectives to previous studies (Laukka, Hammarén, et al. 2022; Morse and Warshawsky 2021), where the digitisation of healthcare services and AI are seen as creating new conditions for future leadership. However, digital services have also been seen in the past as a solution to ensure customers receive quality care in a timely and personalised manner through digital solutions (Al‐Jaroodi et al. 2020; Babaei et al. 2023; Cozzoli et al. 2022). The professionals' perceptions in this study reinforce that future leaders need to be brave to make reforms in digital healthcare, highlighting the importance of leveraging digital transformation to improve health outcomes.

This study provided a novel perspective that to implement effective reforms in practice, future leaders need to know the context in which they are leading, that is, the professionals, customers, other stakeholders and the environment in which services are delivered. Leaders of the future in healthcare organisations need to be at the forefront of the process to ensure the effectiveness of the organisations, and AI might improve it (Ross et al., 2024). As previously studied, there is also a need for policy guidance to address future leadership in order to improve the accessibility and equality of digital healthcare services (Omaghomi et al. 2024). The results of this study emphasised that future leadership requires knowledge management and the use of information as part of decision‐making especially when thinking about middle or top lead. The future frontline leaders were expected to be present and accessible for employees to get the information to transmit it to the leaders at upper levels of the organisation. Using information as part of decision‐making creates value for customers and professionals, as Dal Mas et al. (2023) identified in their study. This study showed that leading in digital healthcare requires competent leaders, and their competence should be strengthened through training, reinforcing the competence needs of leaders identified in previous studies (González‐García et al. 2021). The professionals in this study considered essential as the earlier findings that future leaders need to be trained in digital leadership rather than gaining competence through on‐the‐job training (Brommeyer and Liang 2022; Gjellebæk et al. 2020; Laukka et al. 2023). The professionals' perceptions in this study also confirm the previous findings in the study of Morse and Warshawsky (2021), where they identified the need for leaders to be technically competent.

Professionals in this study perceived that when future leaders are fluent in using digital solutions, it is reflected in a positive experience for professionals. It was found in previous studies that both professionals and leaders need to see working in digital healthcare as positive (Gjellebæk et al. 2020; Laukka, Hammarén, et al. 2022). This study provides insights into the opportunities professionals have identified to strengthen digital positivity. It was found in this study that digital inclusion is significantly strengthened when professionals perceive the benefits of digital solutions as positively impacting their work, which confirms previous findings, as digital solutions aim to smoothen the work of professionals (Kaihlanen et al. 2023). Previous studies have shown that a key factor in committing professionals to use digital solutions is for leaders to act as role models (Laukka et al. 2020).

This study provides novel insights into communication in digital healthcare, highlighting its specific characteristics, as well as the role of humanity and caring practices in leadership. Based on this study, human management can increase well‐being at work, which confirms the findings of previous studies (Juchnowicz and Kinowska 2022; Oleksa‐Marewska and Tokar 2022), underscoring the importance for healthcare organisations to invest in the well‐being of professionals. Previously, Fowler et al. (2021) demonstrated in their study that competent leaders with effective communication are necessary to enhance job satisfaction.

This study highlighted the importance of using leadership to support the well‐being of professionals in digital healthcare. The professionals' perceptions in this study confirm the previous findings, where leaders have also highlighted the need to support professionals' well‐being at work in the future. It has been seen as increasing the attractiveness of healthcare organisations to professionals (Vuorivirta‐ Vuoti et al. 2023). This study found that ensuring adequate resources for adopting the new is essential during all changes. It was seen as supportive of changes to be implemented sustainably. Burgess and Honey (2022) and Vuorivirta‐ Vuoti et al. (2023) also recognised in their studies that adequate resources are needed to implement future practice changes.

This study indicates that professionals require support in developing their digital competencies, enabling them to utilise digital solutions and approaches effectively. Professionals need to be supported in their digital competencies to support their work. Previously, Laukka et al. (2023) stated that leaders need to be able to educate professionals on the use of digital solutions, and the perceptions of professionals confirm this previously stated. This study showed that leaders need to identify and support the competence of different generations. They need to make it visible through collaboration, thus strengthening cohesion and working together. Professionals expect leaders to see the competence of different generations as an asset from which the organisation can benefit. This confirms the findings of Vuorivirta‐ Vuoti et al. (2023) that the leadership of different generations needs to be considered in future leadership to meet the needs of future professionals appropriately. Professionals in this study perceived that younger generations are more comfortable with digital solutions, and previously, Brommeyer and Liang (2022) found that technology is part of the lifestyle of younger generations.

By enabling the involvement of professionals and clients in decision‐making, professionals in this study perceived that digital healthcare services could be designed to meet their needs better. As previously studied, successful healthcare services are built through multi‐professional cooperation and a shared governance culture, which needs to be prioritised in future leadership (Nurmeksela et al. 2021; Omaghomi et al. 2024). Improving involvement in digital healthcare has previously been seen as strengthening professionals' community and belonging (Terkamo‐Moisio et al. 2024). This study provides insights into improving involvement. According to the professionals in this study, involvement and, at the same time, competence development could be strengthened by using talents in the organisation who are professionals working in the organisation. This type of talent utilisation was previously identified in a study by Burgess and Honey (2022).

In this study, professionals perceived limited potential for using AI when leading digital healthcare. University students perceived more opportunities for using AI in future leadership than professionals working in the hospital. The limited potential of AI may be because AI has not been widely adopted in the units of professionals working in university hospitals. The potential of AI in healthcare leadership has been identified in previous studies (Chen et al. 2022; Gonzalez‐Garcia et al. 2024; Petersson et al., 2022). This study perceived that AI's applications are not yet significantly visible to professionals in practice. Or, as Ross et al. (2024) stated, it might be possible that leaders use AI algorithms in decision‐making or when planning work in the background processes that are not visible to the professionals.

### 5.1 Limitations

This study has some limitations. The data were collected from one university hospital, but the multiple perceptions from other organisations were ensured by interviewing the professionals from the university. The participants from the university hospital were recruited from different units and occupations to get broader perceptions about future leadership from the university hospital organisation. There were 5 male and 21 female professionals among the participants. Therefore, gender bias may affect the results, as representatives of different genders may have varying perspectives on issues such as digitalisation in healthcare. This should be considered when applying the presented results to organisations with a higher proportion of male professionals.

Notably, one researcher analysed the data, although it was systematically discussed in the research group. Most of the professionals interviewed (81%) worked in public healthcare, primarily in university hospitals. The Finnish healthcare system is advanced in digitalisation, which may influence professionals' perceptions of future leadership in digital healthcare. Additionally, it was not possible to compare different healthcare roles or hierarchical levels in terms of digital leadership; a comparative analysis could have provided a much richer insight into the phenomenon.

The usage of digital solutions in digital services and leadership can vary between organisations and countries, which needs to be considered when applying the result to other contexts. Due to these aspects, the results of this study may not be transferable to other healthcare organisations or different service systems, although they align with previous studies (Nurmeksela et al. 2021; Vuorivirta‐ Vuoti et al. 2023). It has been 3 years since data collection; healthcare digital solutions and AI have advanced rapidly. Therefore, the results should be interpreted with caution.

## 6 Conclusion

Future leadership will continue to focus on leading people, and humanity will become increasingly critical in digital healthcare. Leaders are expected to be at the forefront of digital solutions, sharing their knowledge with the professionals and nurses they lead with a sense of purpose. Leaders will ensure the involvement of professionals and collaborate to build a future‐focused healthcare system that delivers services sustainably and meets the needs of customers.

Healthcare is often perceived as having gone digital; however, this research reveals that the adoption of digital solutions in healthcare organisations is uneven, influencing how professionals and nurses view the potential of digital solutions in healthcare organisations. In the future, it will be even more critical to identify the reasons why the use of digital solutions varies within an organisation. In this study, the role of AI in leadership within digital healthcare was limited, as professionals and nurses identified little potential for AI in leading digital healthcare initiatives. Further research is needed on the role and purpose of AI in future leadership in digital healthcare, particularly in relation to different healthcare roles or hierarchical levels, as well as the specific competencies future leaders are expected to possess when leading healthcare with AI.

### 6.1 Implications for Nursing Management

Digital healthcare is continually improving over time thanks to the implementation of new digital solutions and the possibilities offered by AI. The culture that future generations will bring to healthcare also impacts healthcare organisations. Future nursing leadership is expected to be more flexible, equal and personalised than ever. The importance of leading digital healthcare holistically and interactively, as well as ensuring the competence of professionals and nurses in a changing environment, is a topical issue for the future. To strengthen the retention and attractiveness of healthcare, it is important to prioritise daily leadership practices that support the well‐being, competence and inclusion of professionals. In the future, competent nurse leaders with the ability to support nursing professionals' digital competence, account for different generations' competencies and needs and strengthen teamwork in a digital work environment will be essential in digital healthcare. There will also be a continuous need to address the leadership in digital healthcare and related education to meet the demands of future professionals, nurses and customers.

## Author Contributions

Conception and design of the manuscript: Marika Määttä, Mira Hammarén, Outi Kanste. Acquisition of data: Mira Hammarén, Suvi Kuha. Analysis and interpretation of data: Marika Määttä, Mira Hammarén, Outi Kanste. Writing the manuscript: Marika Määttä, Mira Hammarén, Suvi Kuha, Outi Kanste. Commenting on the manuscript: Marika Määttä, Mira Hammarén, Suvi Kuha, Outi Kanste.

## Ethics Statement

Permission was obtained from the target hospital and university to conduct the research. According to Finnish legislation, this study did not require Research Ethics Committee approval since the interviews did not concern patients or minors, and the interviews did not intervene in physical or mental impunity.

## Consent

The authors have nothing to report.

## Conflicts of Interest

The authors declare no conflicts of interest.

## Supporting information


**Data S1:** COREQ (COnsolidated criteria for REporting Qualitative research) checklist.

**Table S1:** Healthcare professionals’ direct quotes by main category.

## Data Availability

Research data are not shared.
