# Supplementary material for: Healthcare Professionals' Perceptions of Future Leadership in Digital Healthcare: A Qualitative Study
Source: J Adv Nurs. 2025 May 8;82(2):1482–97. doi: 10.1111/jan.17035 (PMC12810602; doi:10.1111/jan.17035)
Supplement: Supplementary file 2 — Table S1: Healthcare professionals’ direct quotes by main category. [file JAN-82-1482-s002.docx]

Supplementary Table 1. Healthcare professionals' direct quotes by main category.

| **Main category** | **Original expressions** |
| --- | --- |
| Building a future-oriented healthcare | Leaders would involve those who do the work and those who know most about the practicalities of doing the work in the development process. And, in (evaluating) whether the new service is needed and how to make it work. But also in planning the implementation. (Interviewee 12) |
|  | Especially in larger organisations like this one, we can't just think about our unit or department. We need to think more holistically about the patient care process. (Interviewee 7) |
|  | The leader should have the time and expertise to support the employee's adaptation and introduction to the new services. (Interviewee 12) |
|  | It would be worth taking the time to look at it and, in a way, take the attitude that this is the future, and we must go into this (digital environment). That we are able to maintain and secure our healthcare services. So, in my opinion, a change of attitude is the key. (Interviewee3) |
|  | Well, first of all, leaders should have a low threshold for this digital development. They should have the will to push things forward and make them more accessible to staff. (Interviewee 6) |
|  | In other words, take it one step at a time rather than all at once. This could make it easier to introduce new features. (Interviewee 10) |
|  | Well, of course, that the leader has an understanding of the entire thing and the things that make up the entity. (Interviewee 2) |
|  | If we are talking about HR management at the front line, the leader needs to have an understanding of the wider picture of the organization. (Interviewee 2) |
|  | Of course, we must always be aware that other professionals are also part of the patient's care, and their perspectives should also be considered. (Interviewee 7) |
| Strengthening a digitally minded organisational culture | The leader has a pacifier role. So that when people are panicking, the leader can get them excited about it (the change) and create this "you'll learn" atmosphere. (Interviewee 1) |
|  | I think it's really important in all of this that the leader is supportive. That includes positivity, but that attitude is encouraging. The kind of "you'll get through it". That's what keeps you going, no matter what you're up against. (Interviewee 1) |
|  | If someone feels that something is difficult, let's get someone who is better at it and knows how to do it to work as a pair with them for a while. To support them and help them with the new thing, so that will help. (Interviewee 1) |
|  | That in leadership would be used to consider that, hey, it now seems that we can't do anything with this (digital solution), so let's not take it to us.  (Interviewee 4) |
| Being interactive in a digital environment | The leader must be passionate about the subject and have a desire to spread knowledge, share knowledge and enable knowledge to be shared. (Interviewee 1) |
|  | The leader must also point out that it (digitalization) is useful in some way for our work. And that they can also tell us what benefits it might have in the future, even if it seems a bit difficult at first. (Interviewee 11) |
|  | So that everyone has access to the same information at the same time. When it is easy to get a bit too much information when it is spread across many different platforms. (Focus group 2 Interviewee 2) |
|  | It occurs to me that the role of the frontline leader is all about communication. And leading the communication. (Focus group 2 Interviewee 4) |
|  | I think it is the responsibility of the frontline leaders to make sure that the information flows from the top down to the frontline employees. And also, for information to flow upwards from the employees. The frontline leader enables this and provides the right forums for it. (Focus group 2 Interviewee 3) |
|  | At the very least, I wonder if my concern or issue is really being heard. And do I feel that they are trying to do something about it? More importantly, how do you get that feeling of being heard, when communicating digitally as compared to face-to-face interaction? (Focus group 3 Interviewee 1) |
| Leading sustainably in digital healthcare | With leadership it's important to allow us to interact face-to-face, whether it's in teams or wherever. (Focus group 3, interviewee 3) |
|  | I think it's important that when it comes to managing people, the manager has to be very attentive, take people into account and listen to everyone. So, in a digital environment, it is even more important that the leader gives the opportunity to speak and participate in the discussion. (Focus group 3 Interviewee 1) |
|  | When working live, you are able to notice the little nuances, people's gestures and facial expressions and body language. And when that's missing in the digital environment, I think the leader needs very precise ears. (Focus group 2 Interviewee 1) |
|  | Digitalisation allows us to operate globally. Therefore, understanding and being competent in multiculturalism requires special skills that the leaders may not have needed before. (Focus group 2 Interviewee 2) |
|  | Monitoring wellbeing in the workplace can be challenging in these digital services. (Focus group 1 Interviewee 2) |
| Leading expertise in digital healthcare | We have a really wide age range in the workplace, with a variety of learners. I think it is the responsibility of the frontline leader to make sure that the whole team and each member of the team has enough time to adapt to these new changes. (Focus group 2 Interviewee 3) |
|  | The leader needs to understand the challenge of digital solutions, which can also be due to lack of time, the age of the employee or how comfortable the employee is to use different digital services in general. The leader needs to understand that it's not easy for everyone. (Interviewee 12) |
|  | When employees are attending training, the leader should make time for them to share with another team what they have learned. It is also part of leadership to be interested in what kind of training the employees have attended. (Interviewee 2) |
|  | The leaders can't just assume that if they give the tasks to younger employees, they'll be much better at it. They also must support the employee who is not so good at it. I think that's a demand for a leader. (Interviewee 1) |
|  | Leaders need to be aware that we all have our places and our roles in the working community. The leaders need to recognise the importance of new visions and the topical things that the younger employees have learned from the school lately. They have learned a lot of new things and ways of doing things at school. Whereas the role of the older employees is to bring life experience and a calmness to the work community. (Interviewee 3) |
|  | In particular, the leader needs to recognise and consider that there are people of different ages to be led. (Focus group 2 Interviewee 4) |
| Leading collaboratively in digital healthcare | The key to leadership is sensitivity and the ability to listen to what the employees really think about the services they provide. In other words, what kind of services, applications and technologies are needed. In other terms, the leaders need to listen to the needs of the working team rather than create the needs themselves. (Focus group 1 Interviewee 1) |
|  | The use of digital connectivity in leadership makes processes more efficient. (Focus group 4 Interviewee 3) |
|  | It is important that the leaders do not just lead from the top to down in a bureaucratic way like "you have to do this and that". Also, the employees from bottom to top can really bring in their own views and new things that the services require. And especially what kind of development the services need. (Focus group 4 Interviewee 3) |
|  | Leaders must be able to connect and communicate effectively with those they lead and build trust through digital solutions. (Focus group 1 Interviewee 4) |
| Using artificial intelligence in leadership in digital healthcare | Artificial intelligence information systems can certainly provide a great deal of information to support leadership, but you also need to know how to use the information. (Focus group 1 Interviewee 3) |
|  | For example, in my own work there are very complex and challenging work capacity issues. So, it seems difficult to know whether and how well artificial intelligence could be used in any of these areas in the social and health sector. (Focus group 4 Interviewee 2) |
|  | The risk or challenge of artificial intelligence depends on what the organization wants from artificial intelligence in leadership. Do they want artificial intelligence to work in a humane and inclusive way? Or do they use it to design work to discover economic efficiency or production efficiency? (Focus group 1 Interviewee 1) |
|  | Perhaps I see a small threat or risk in the loss of that human touch and human leadership (as the use of artificial intelligence in leadership increases). (Focus group 1 Interviewee 1) |
